# Artificial intelligence and pelvic fracture diagnosis on X-rays: a preliminary study on performance, workflow integration and radiologists' feedback assessment in a spoke emergency hospital

**DOI:** 10.1016/j.ejro.2023.100504

**Published:** 2023-07-06

**Authors:** Francesca Rosa, Duccio Buccicardi, Adolfo Romano, Fabio Borda, Maria Chiara D’Auria, Alessandro Gastaldo

**Affiliations:** a Diagnostic Imaging Department, San Paolo Hospital, ASL 2, via Genova 30, Savona, Italy; b Italian Society of Medical and Interventional Radiology (SIRM), SIRM Foundation, Milan, Italy; c Department of Health Sciences (DISSAL) – Radiology Section, University of Genoa, 16132 Genoa, Italy

**Keywords:** Artificial intelligence, Plain radiograph, Fracture, Computed tomography

## Abstract

**Purpose:**

The aim of our study is to evaluate artificial intelligence (AI) support in pelvic fracture diagnosis on X-rays, focusing on performance, workflow integration and radiologists’ feedback in a spoke emergency hospital.

**Materials and methods:**

Between August and November 2021, a total of 235 sites of fracture or suspected fracture were evaluated and enrolled in the prospective study. Radiologist’s specificity, sensibility accuracy, positive and negative predictive values were compared to AI. Cohen's kappa was used to calculate the agreement between AI and radiologist. We also reviewed the AI workflow integration process, focusing on potential issues and assessed radiologists’ opinion on AI via a survey.

**Results:**

The radiologist performance in accuracy, sensitivity and specificity was better than AI but McNemar test demonstrated no statistically significant difference between AI and radiologist’s performance (*p* = 0.32). Calculated Cohen’s K of 0.64.

**Conclusion:**

Contrary to expectations, our preliminary results did not prove a real improvement of patient outcome nor in reporting time but demonstrated AI high NPV (94,62%) and non-inferiority to radiologist performance. Moreover, the commercially available AI algorithm used in our study automatically learn from data and so we expect a progressive performance improvement. AI could be considered as a promising tool to rule-out fractures (especially when used as a “second reader”) and to prioritize positive cases, especially in increasing workload scenarios (ED, nightshifts) but further research is needed to evaluate the real impact on the clinical practice.

## 1 Introduction

One of the most common causes of Emergency Department (ED) visits is bone fractures, and X-ray is the first-line imaging technique for the diagnosis of these lesions.

Reporting trauma X-rays is a demanding task that requires radiologic expertise, despite the current shortage of radiologists [1], [2].

Diagnostic errors are indicators of inadequate patient care and can lead to variable consequences, from minimal to life-threatening ones. Diagnostic delays caused by interpretative errors may lead to delayed treatments, increased surgical risks, and poor outcomes. Recent studies on patients' complaints revealed that 75% are due to interpretative mistakes and consequent incorrect diagnoses. Fracture misdiagnosis is one of the most frequent diagnostic errors and the major reason for paid malpractice claims: detecting thin fracture lines can be extremely challenging, and anatomic variants or previous traumas may be misinterpreted.

In this clinical scenario, artificial intelligence (AI) solutions could have an important role in decreasing the percentage of fracture misdiagnosis [1], [3].

The risk of missing a subtle fracture increases according to physician fatigue (e.g., during a night shift or long busy day), even in the case of experienced radiologists [4]. Therefore, an AI solution that offers a second opinion by highlighting suspicious areas on images may allow standardizing quality and reducing errors, leading to more efficient interpretations. Considering recent advances in deep learning (DL) and computer vision, AI may play a pivotal role in this field [2].

Time constraints/efficiency, error avoidance/minimization, and workflow optimization are the most significant drivers for the development of AI as a tool in the healthcare setting.

The development of an effective AI system for image reporting could reduce the time spent reviewing images by 20%. This time can be spent on non-automatable tasks such as providing personalized patient care and more complex tasks where human input is crucial [5].

AI, machine learning (ML), DL, and convolutional neural networking (CNN) are the keywords, and they are interconnected as follows. AI is defined as computer systems able to perform tasks that mimic human intelligence. ML, a subfield of AI, allows a machine to learn and improve from experience, independently of human action. DL, a more specialized subfield of ML, analyses more data sets, transforming algorithm inputs into outputs through computational models such as deep neural networks [2]. CNN is an evolutionary computational technique of DL, made up of multilayer perceptrons. Multilayer perceptrons consist of fully connected networks where each “neuron” in one layer is connected to all “neurons” in the next layer.

The connectivity pattern between neurons and the organization of the animal visual cortex inspired CNN development. Like the receptive field, each cortical neuron reacts to stimuli only in a restricted region of the visual field.

The entire visual field is covered thanks to the partial overlap of the different neuron's receptive fields. CNNs apply relatively little pre-processing in contrast with the other image classification algorithms. The network learns to optimize the filters (or kernels) through automated learning, and not through hand-engineered filters such as traditional algorithms. This independence from previous knowledge and human intervention in feature extraction is one of the major advantages [6], [7], [8].

The aim of our study is to evaluate prospectively and in a clinical environment the AI performance in fracture diagnosis on X-rays We decided to evaluate AI performance only in pelvic fractures diagnosis due to their important clinical impact and complexity: unstable pelvic fractures can be fatal due to pelvic haemorrhage and can require prompt management. Moreover, their diagnosis can be challenging due to overlapping structures.

Our work also focuses on workflow integration issues and preliminary radiologists’ feedback in a spoke emergency hospital.

## 2 Materials and methods

This prospective study was approved by the institutional review board approval (n 455 17/6/21) and informed consent was waived because analysis used anonymous data.

### 2.1 Study population

This was a prospective study performed at a single medical centre (spoke emergency hospital) from August to November 2021. After obtaining institutional review board approval, patients who presented to our ED with a pelvic trauma, underwent pelvis X-rays and were enrolled in our study.

In the first steps of our study, AI aided diagnosis was required by radiologists on a voluntary basis. Poor quality exams, precluding human interpretation, were excluded from the study.

A total of 223 patients were included and a total of 235 sites of fracture or suspected fracture were evaluated. 7 patients had multiple fractures. The whole ED radiology staff (26 radiologists) participated in the study.

### 2.2 AI software and AI workflow integration

**Fig. 1:**
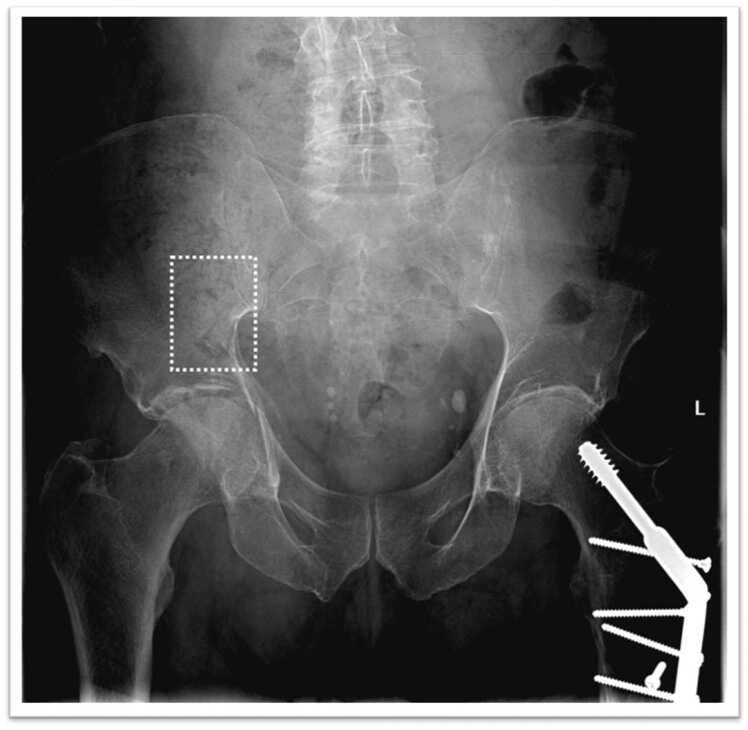
Left inferior pubic ramus - doubt fracture (AI processed image).

**Fig. 2:**
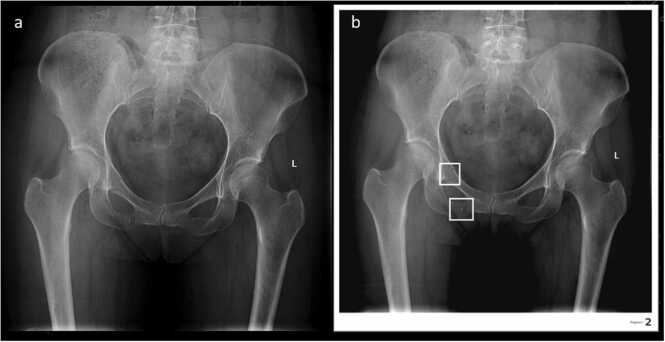
Right superior and inferior pubic ramus – fracture: A) native, B) AI processed (native).

We used a commercially available CE class IIA AI solution: a deep CNN based on the “Detectron 2” framework able to detect and localize fractures on native resolution digital radiographs, integrated into a radiology software as a diagnostic aid, highlighting each region of interest with a box and providing a confidence score about the presence of a fracture within it (solid line for highly suspected and dashed line for doubt), as shown in Fig. 1, Fig. 2.

Each exam was at first interpreted by ED radiologist, blinded to AI results (unaided diagnosis). AI processed images were subsequently retrieved for AI aided diagnosis.

### 2.3 Reference standard

Final diagnoses were established by a Senior Radiologist with 10 years-experience in Muscoloskeletal imaging. 37 AI- Radiologist discordant cases and concordant negative cases with a positive/doubtful clinical examination underwent Computed Tomography (CT).

**Fig. 3:**
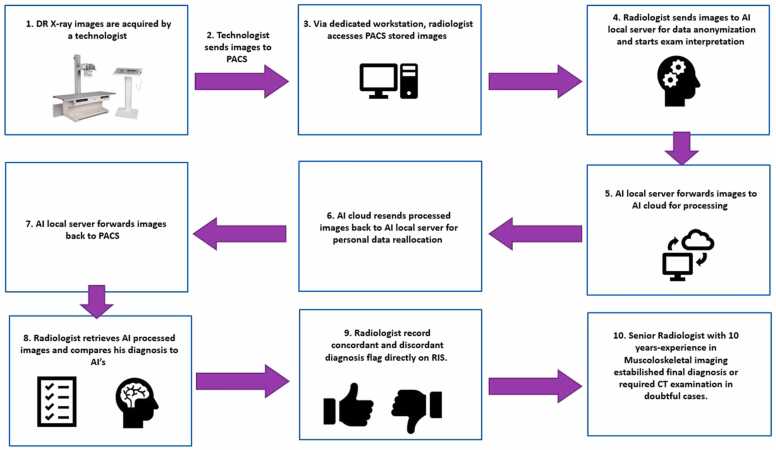
Scheme of AI software integration in ED radiology workflow.

Scheme of AI software integration in ED radiology workflow is showed in Fig. 3.

### 2.4 Statistical analysis

Statistical analyses were performed using MedCalc software calculator. We compared AI and radiologist performance using the accuracy, sensitivity, specificity, and 95% confidence intervals (CIs) of each parameter.

The McNemar test was used to evaluate the accuracy, sensitivity, and specificity non-inferiority of AI compared to radiologists.

The kappa coefficient was calculated between the AI and radiologist diagnoses.

## 3 Results

The overall accuracy, sensitivity, specificity positive and negative likelihood ratio of AI and radiologists are shown in Table 1.

**Table 1:** Statistical analysis.

Statistic	Specificity(%)		95% CI	Sensitivity (%)	95% CI	Accuracy (%)	95% CI	Positive likelihood ratio	95% CI	Negative likelihood ratio	95% CI
Radiologists	96,88	93.32 - 98,84		79,07	63,96- 89,96	93.62	89.69–96.38	25.30	11.34–56.45	0.22	0.12–0.39
AI software	91.67	86.82–95.16		76.74	61,37 -88,24	88.94	84.21–92.64	9.21	5.6–15.14	0.25	0.15–0.44

**Fig. 4:**
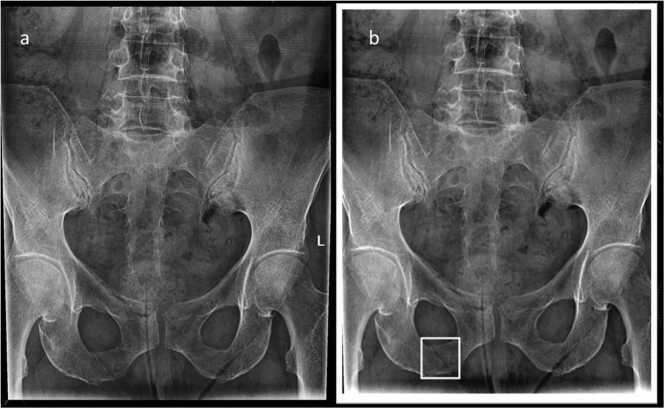
Right inferior pubic ramus – FP fracture (skin fold): A) native, B) AI processed.

AI and radiologists' specificity were comparable (respectively 91.67% and 96,88%) with respectively 16 and 6 false positive (FP), Fig. 4.

**Fig. 5:**
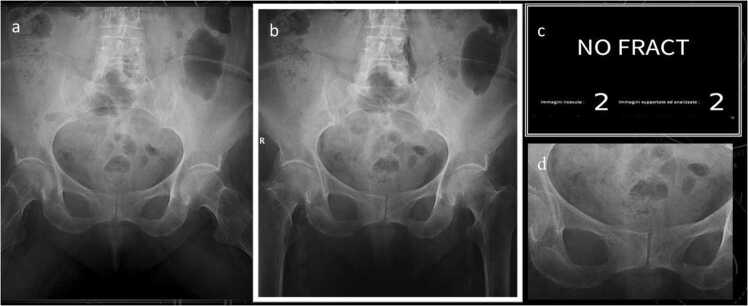
Right superior pubic ramus – AI FN fracture (native) A) native, B) AI processed.

AI sensitivity was lower than radiologist (76,74% versus 79.07%) with respectively 10 and 9 false negative (FN), Fig. 5. Positive Predictive Value (PPV) and NPV were respectively 85% and 95,38% for radiologists and 67,35% and 94,62% for AI.

The radiologist performance in accuracy, sensitivity and specificity was better than AI but McNemar test demonstrated no statistically significant difference between AI and radiologist’s performance (*p* = 0.32).

235 sites of suspect fracture were included and 210 presented with concordant reports (% of agreement was 89.4%): 30 (12.8%) with fractures and 180 (76.6%) with no fractures.

There were 25 (10.6%) discordant reports: 15 (6.4%) negatives for AI and positives for radiologist and 10 (4.2%) negatives for radiologist and positives for AI.

The Kappa coefficient between AI and radiologist was 0.641 (95% CI from 0.512 to 0.770), which means a substantial agreement.

At the end of the study, all radiologists were asked to fill a 5-point response Linkert scale survey for feedback assessment (results are reported in Table 2).

**Table 2:** Radiologist’s feedback survey.

Radiologist's Feedback survey	Rating scale				
Responders specify their level of agreement to a statement in five points:	1 Strongly agree (%)	2 Agree (%)	3 Neither agree nor disagree (%)	4 Disagree (%)	5 Strongly disagree (%)
Images sending process is quick and easy	11.11	33.33	22.22	33.33	0.00
Image processing times are acceptable	11.11	33.33	33.33	22.22	0.00
AI increases your diagnostic confidence	0.00	0.00	22.22	0.00	77.78
AI increases fracture detection in overload situations	0.00	0.00	11.11	11.11	77.78
Other Specialists should be able to view the AI processed images	0.00	22.22	33.33	22.22	22.22
Patients should be able to view the AI processed images	11.11	22.22	33.33	0.00	33.33
AI reduces malpractice risks	0.00	11.11	33.33	22.22	33.33
AI may increase malpractice risk	33.33	44.44	22.22	0.00	0.00
I am satisfied of AI support in fracture detection	0.00	22.22	66.67	11.11	0.00

## 4 Discussion

Pelvic fracture diagnosis and treatment pose significant challenges. A previous study indicated that X-rays have a sensitivity of only 78% in acute trauma patients [9].

In our testing, the AI software exhibited a good accuracy rate (88.94%) in detecting pelvic fractures when compared to the detection accuracy of radiologists (93.62%).

Our prospective study did not demonstrate a tangible improvement in patient outcomes or reporting time. However, we did establish the high NPV of AI (94.62%) and its non-inferiority to radiologists' performance. Considering that CNN can enhance its performance, this type of AI software holds promise as a tool to reduce misdiagnoses.

Delayed diagnosis of pelvic fractures, particularly unstable ones, can lead to a poor prognosis and increased risk of death: AI assists in promptly classifying a radiograph as positive or negative, also enabling prioritization of positive cases within the worklist.

A previous prospective study on this topic was published; however, AI was not integrated into the clinical workflow [10]. Oakden-Rayner et al. proposed an external validation dataset for a deep learning system to detect proximal femoral fractures, which was evaluated prospectively but not in a clinical environment [10].

To the best of our knowledge, this is one of the first true prospective studies to apply AI in a real-time scenario [11].

During the early stage of our study, we encountered the following issues:

• Integration of AI hardware and software into the hospital's informatics network and our ED clinical workflow.
• Manual transmission of X-ray images to the AI server.
• Long processing time for AI (minutes).

All these issues were promptly resolved within the first week. RIS-PACS and AI engineers collaborated to achieve optimal network integration, automatic image transmission, and a drop in processing time to seconds.

Interpreting pelvis X-rays can be challenging due to artifacts caused by incorrect positioning during image acquisition (especially for elderly and severely painful patients), overlapping anatomical structures (e.g., skin folds, stool), and bowel meteorism. These artifacts result in interpretation difficulties for both radiologists and AI.

Previous study demonstrated that AI performs better in fracture detection in anatomical areas with fewer artifacts and overlapping structures: highest sensitivity was demonstrated on shoulder/clavicle x-rays and lowest sensitivity in ribcage ones [12].

Our data on pelvic fracture detection showed that radiologists performed better than AI, although the difference was not statistically significant. AI sensitivity was lower than that of radiologists, but AI and radiologists had comparable specificity (91.67% and 96.88%, respectively). Additionally, at least 5 out of 16 AI FP cases were easily recognized by radiologist review, including 3 skin fold artifacts and 2 old fractures (where comparisons with previous radiographs were crucial). We expect these minor errors to be corrected by AI's dynamic self-paced function.

Our study had some limitations, such as a small patient cohort, short recruitment and observation periods, and the inapplicability of CT examination as a reference standard for all cases.

The feedback assessment from radiologists produced conflicting results. Most colleagues were likely sceptical about this new technology, and some of the older ones may have feared being replaced by AI in the future.

Concerning the role of AI in malpractice risks, many radiologists perceive AI as a "black box" where inputs and outputs are clear, but the intermediate process remains unclear. This lack of transparency could lead to distrust and resistance. The need for Explainable AI is an emerging research topic focused on understanding how AI systems make their choices [13].

Only a small number of colleagues currently acknowledge the usefulness and value of AI as a supportive tool in fracture detection, particularly in situations of overload, such as night shifts. These colleagues believe that AI has the potential to enhance radiologists' performance, improve patients' management, streamline the medical decision-making process, and enhance the overall quality of healthcare [14].

It is widely recognized that new technologies have the capacity to enhance the quality, efficiency, and safety of healthcare devices. However, introducing a new informatics tool can be a delicate process in certain healthcare settings, as it may entail new risks and elicit individual concerns [15].

## 5 Conclusion

To the best of our knowledge, this study represents one of the first prospective investigations to apply AI in real-time clinical practice and discuss the integration challenges within the clinical workflow.

Contrary to our initial expectations, the preliminary results did not demonstrate a significant improvement in patient outcomes or reporting time. However, the study did highlight NPV of AI (94.62%) and its non-inferiority to radiologist performance.

Furthermore, the commercially available AI algorithm utilized in our study has the capability to continuously learn from data, which suggests that its performance could progressively improve over time.

AI shows promise as a tool for ruling out fractures, particularly when used as a "second reader," and for prioritizing positive cases, especially in scenarios of increased workload, such as emergency departments and night shifts. Nevertheless, further research is necessary to evaluate the actual impact of AI on clinical practice.

## Consent for publication

Institutional review board approval was obtained and the need for written informed consent was waived because the manuscript does not contain any patient data.

## Funding

The authors state that this work has not received any funding.

## CRediT authorship contribution statement

All authors contributed to data acquisition or data analysis/interpretation, Material preparation, data collection and analysis were performed by Rosa Francesca, Duccio Buccicardi, Fabio Borda and Gastaldo Alessandro. The first draft of the manuscript was written by Rosa Francesca and Duccio Buccicardi and all authors commented on previous versions of the manuscript. All authors read and approved the final manuscript.

## Declaration of Competing Interest

The authors declare that they have no known competing financial interests or personal relationships that could have appeared to influence the work reported in this paper.

## References

[1] Duron L., Ducarouge A., Gillibert A., Lainé J., Allouche C., Cherel N., Zhang Z., Nitche N., Lacave E., Pourchot A., Felter A., Lassalle L., Regnard N.E., Feydy A. (2021). Assessment of an AI Aid in Detection of Adult Appendicular Skeletal Fractures by Emergency Physicians and Radiologists: A Multicenter Cross-sectional Diagnostic Study. Radiology.

[2] AlGhaithi A., Al Maskari S. (2021). Artificial intelligence application in bone fracture detection. J. Musculoskelet. Surg. Res..

[3] Pinto A., Reginelli A., Pinto F., Lo Re G., Midiri F., Muzj C., Romano L., Brunese L. (2016). Errors in imaging patients in the emergency setting. Br. J. Radiol..

[4] Krupinski E.A., Schartz K.M., Van Tassell M.S., Madsen M.T., Caldwell R.T., Berbaum K.S. (2017). Effect of fatigue on reading computed tomography examination of the multiply injured patient. J. Med Imaging (Bellingham)..

[5] Raine C., McConnell J., Hughes C., Bond R., McFadden S. (2021). Artificial intelligence for diagnosis of fractures on plain radiographs: A scoping review of current literature. Intell. -Based Med..

[6] Hubel D.H., Wiesel T.N. (1968). Receptive fields and functional architecture of monkey striate cortex. J. Physiol..

[7] Fukushima K. (1980). Neocognitron: a self-organizing neural network model for a mechanism of pattern recognition unaffected by shift in position. Biol. Cyber.

[8] Matsugu M., Mori K., Mitari Y., Kaneda Y. (2003). Subject independent facial expression recognition with robust face detection using a convolutional neural network. Neural Netw..

[9] Obaid A.K., Barleben A., Porral D., Lush S., Cinat M. (2006). Utility of plain film pelvic radiographs in blunt trauma patients in the emergency department. Am. Surg..

[10] Oakden-Rayner L., Gale W., Bonham T.A., Lungren M.P., Carneiro G., Bradley A.P., Palmer L.J. (2022). Validation and algorithmic audit of a deep learning system for the detection of proximal femoral fractures in patients in the emergency department: a diagnostic accuracy study. Lancet Digit Health.

[11] Dupuis M., Delbos L., Veil R., Adamsbaum C. (2022). External validation of a commercially available deep learning algorithm for fracture detection in children. Diagn. Inter. Imaging.

[12] Oppenheimer J., Lüken S., Hamm B., Niehues S.M. (2023). A prospective approach to integration of AI fracture detection software in radiographs into clinical workflow. Life (Basel).

[13] Yang G., Ye Q., Xia J. (2022). Unbox the black-box for the medical explainable AI via multi-modal and multi-centre data fusion: A mini-review, two showcases and beyond. Inf. Fusion.

[14] Giordano C., Brennan M., Mohamed B., Rashidi P., Modave F., Tighe P. (2021). Accessing Artificial Intelligence for Clinical Decision-Making. Front Digit Health.

[15] Mytton O.T., Velazquez A., Banken R., Mathew J.L., Ikonen T.S., Taylor K., Painter F., Jean-Baptiste R., Poon A., Ruelas E. (2010). Introducing new technology safely. Qual. Saf. Health Care.

